# A variant of the castor zinc finger 1 (*CASZ1*) gene is differentially associated with the clinical classification of chronic venous disease

**DOI:** 10.1038/s41598-019-50586-2

**Published:** 2019-09-30

**Authors:** Gregory T. Jones, Judith Marsman, Luba M. Pardo, Tamar Nijsten, Marianne De Maeseneer, Vicky Phillips, Chi Lynch-Sutherland, Julia Horsfield, Jolanda Krysa, Andre M. van Rij

**Affiliations:** 1 Departments of Surgery, Dunedin School of Medicine, University of Otago, PO Box 56, Dunedin, 9054 New Zealand; 2 Department of Dermatology, Erasmus MC, Rotterdam, Dr. Molewaterplein 40, 3015 GD Rotterdam, The Netherlands; 3 Department of Pathology, Dunedin School of Medicine, University of Otago, PO Box 56, Dunedin, 9054 New Zealand; 4 Maurice Wilkins Centre for Molecular Biodiscovery, The University of Auckland, Private Bag, 92019 Auckland, New Zealand

**Keywords:** Functional genomics, Genetic association study

## Abstract

Recent reports have suggested a reproducible association between the rs11121615 SNP, located within an intron of the castor zinc finger 1 (*CASZ1)* gene, and varicose veins. This study aimed to determine if this variant is also differentially associated with the various clinical classifications of chronic venous disease (CVD). The rs11121615 SNP was genotyped in two independent cohorts from New Zealand (n = 1876 controls /1606 CVD cases) and the Netherlands (n = 1626/2966). Participants were clinically assessed using well-established CVD criteria. The association between the rs11121615 C-allele and varicose veins was validated in both cohorts. This was strongest in those with higher clinical severity classes and was not significant in those with non-varicose vein CVD. Functional analysis of the rs11121615 variant demonstrated that the risk allele was associated with increased enhancer activity. This study demonstrates that the *CASZ1* gene associated C-allele of rs11121615 has a significant, reproducible, association with CVD (CEAP C ≥ 2 meta-odds ratio 1.31, 95% CI 1.27–1.34, *P* = 1 × 10^−98^, *P*Het = 0.25), but not with non-varicose vein (CEAP C1, telangiectasia or reticular veins) forms of venous disease. The effect size of this association therefore appears to be susceptible to influence by phenotypic heterogeneity, particularly if a cohort includes a large number of cases with lower severity CVD.

## Introduction

Chronic venous disease is common^1^ and is associated with a significant socioeconomic burden^2^, accounting for an estimated 1–2% of total health budget costs in Western Europe and the United States of America^3^. Family-based studies suggest that CVD has a strong genetic risk component^4–6^. Although a large number of candidate gene-based studies have been previously undertaken, these have failed to produce robust genetic associations^7^. In recent years several genome-wide association studies (GWAS) have been conducted with a specific focus on varicose veins of the lower limbs. The first of these was conducted by 23andMe, examining European participants with self-declared varicose veins. This work was presented in poster form at the 64th Annual Meeting of the American Society of Human Genetics in 2014^8^. This was followed by a GWAS examining more clinically verified cohorts of German participants^9^. Interestingly, there appeared to be little overlap between the results of these two GWAS. Very recently Shadrina and colleagues performed a validation of the top hits from both of these two GWAS, using two independent cohorts from Russia and the United Kingdom (UK Biobank)^10^. By far the strongest association, both in terms of effect size and statistical power, was with rs11121615, initially identified in the 23andMe study. This single nucleotide polymorphism (SNP) is located within an intron of the castor zinc finger 1 (*CASZ1)* gene however, to date, the functional consequence of this variant is unclear.

We therefore undertook a validation study, in two independent cohorts from New Zealand (NZ) and the Netherlands (NL), to compare the association of this risk variant with different clinical classifications of venous disease. In addition, we performed functional analyses to determine the potential biological mechanism(s) through which the risk allele may be able to influence disease pathobiology.

## Results

The demographics of the NZ and NL cohorts are shown in Table 1. The proportion of participants with CEAP C1 disease reflected the differing designs of the two studies (NZ: selected cases referred for clinical venous assessment, NL: a population-based cohort study). Accordingly, over half (57.5%) of the NL case cohort were in the CEAP C1 group while only 6% of the NZ cases had this clinical classification.

**Table 1:** Demographics of the New Zealand and Netherlands cohorts divided by chronic venous disease clinical classification.

	Controls, CEAP C0	Self-declared VV	CEAP C1	CEAP C2	CEAP C3	CEAP C4	CEAP C5-6	Varicose veins CEAP ≥ C2
**New Zealand, n**	1876	564	97	535	60	202	148	1509
Age	68.8 (7.6)	67.7 (9.5)	71.2 (10.5)	60.4 (14.7)	63.3 (12.0)	60.8 (11.8)	63.0 (12.6)	64.3 (12.3)
sex (% male)	64.6	50.0	56.4	38.7	46.6	55.3	56.7	47.7
**The Netherlands, n**	1626	—	1704	925	268	62	7	1262
age	68.4 (9.6)	—	69.4 (9.8)	71.9 (9.7)	75.3 (9.1)	76.0 (10.9)	67.5 (14.1)	72.8 (9.8)
sex (% male)	53.4	—	34.2	39.7	38.4	40.3	57.4	39.5

Participants were characterised using the clinical classification matrix of the CEAP chronic venous disease criteria, except a group of self-declared NZ participants, who were visually inspected to confirm varicose veins and exclude those with CEAP C1.

CEAP clinical classifications; (C0) No evidence of venous disease, (C1) telangiectasia or reticular veins, (C2) uncomplicated varicose veins, (C3) edema, (C4) Skin changes ascribed to venous disease (pigmentation or eczema, lipodermatosclerosis), (C5) healed or (C6) active venous ulcer. Age is represented as mean (standard deviation).

### The association between rs11121615 and chronic venous disease

The rs11121615 SNP was in Hardy-Weinberg equilibrium in both the NZ (cases *P* = 0.86 and controls *P* = 0.10) and NL (cases *P* = 0.22 and controls = 0.07) cohorts.

**Figure 1:**
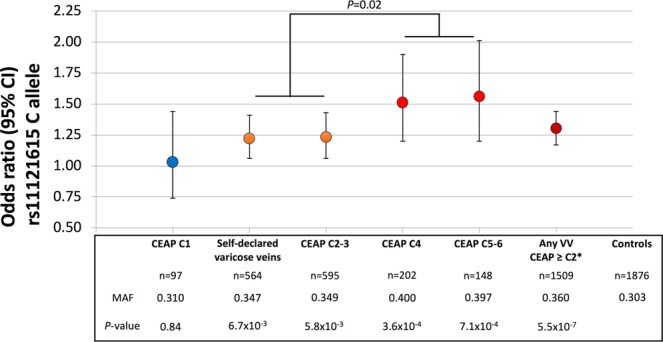
rs11121615 C-allele (*CASZ1*) association with chronic venous disease in the New Zealand cohort. *The ‘Any VV’ group includes those with clinically classified (CEAP ≥ C2) and the self-declared (but visually verified) varicose veins.

**Figure 2:**
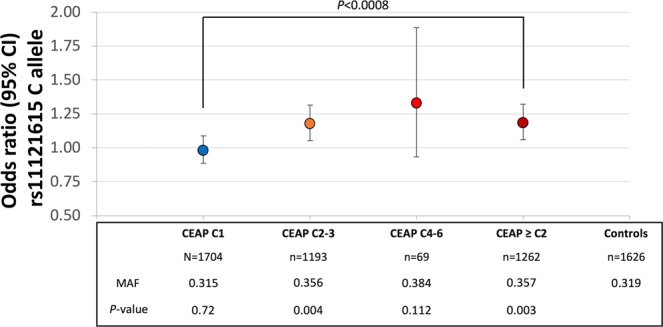
rs11121615 C-allele (*CASZ1*) association with chronic venous disease in the Netherlands study.

The rs11121615 minor allele frequencies (MAF) for both cohorts are summarized in Figs 1 and 2. The frequency of the minor C-allele was similar in the control cohorts from both NZ and NL (MAF 0.30 and 0.32 respectively, *P* = 0.15). There was no statistical difference in the allele frequencies between controls and case participants with CEAP C1 venous disease in either study.

In both cohorts, those with varicose veins (CEAP ≥ C2 or self-declared but visually verified varicose veins) had a higher C-allele frequency than either controls or participants with CEAP C1. In the NL cohort, CEAP C1 participants had a significantly (*P* < 0.0008) lower C-allele frequency than those with a CEAP ≥ C2 (Fig. 2). Given that CEAP C1 classified venous alterations are not considered to be varicose veins^11^, we then excluded such individuals from the subsequent association analysis. The unadjusted odds ratio for the C-allele being associated with varicose veins (CEAP ≥ C2 or visually verified varicose veins) were 1.30 (95% CI 1.17–1.44, *P* = 5.5 × 10^−7^) and 1.18 (1.06–1.32, *P* = 2.5 × 10^−3^) in the NZ and NL cohorts (Figs 1 and 2 respectively and Supplementary Table 2). These associations remained significant after including age and sex as covariates (1.27, 1.14–1.42, *P* = 9.9 × 10^−6^ and 1.17, 1.04–1.31, *P* = 0.01 respectively).

**Figure 3:**
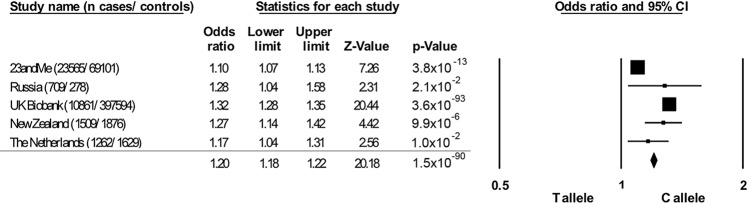
rs11121615 C-allele (*CASZ1*) meta-analysis association with chronic venous disease. Both the NZ and NL cohorts consisted of chronic venous disease cases with CEAP ≥ C2. Case control status in the 23andMe cohort was self-declared (unverified). The Russian cases and controls were confirmed by clinical examination (CEAP ≥ C2), while the UK Biobank case status was electronically extracted from ICD10 clinical modification codes (I83; varicose veins of the lower extremity). Results from all cohorts were adjusted for age and sex.

A meta-analysis was performed using the major T-allele as the reference and minor C-allele as the risk variant. The odds ratios from all contributing cohorts were adjusted for age and sex. The NZ and NL C-allele associations in those with varicose veins and more advanced CVD (by excluding those with CEAP C1) were analyzed in a fixed effect model meta-analysis with the previously reported 23andMe, Russian and United Kingdom associations. The resulting odds ratio was highly statistically significant (Fig. 3, odds ratio 1.20, 95% CI 1.18–1.22, *P* = 1.5 × 10^−90^). Despite consistent directions of effect across all cohorts there was significant heterogeneity in the fixed effect model (I^2^ 95.8, *P*Het < 1 × 10^−8^). This was driven primarily by the inclusion of the 23andMe cohort, with the meta odds ratio rising to 1.31 (95% CI 1.27–1.34, *P* = 9.0 × 10^−99^) and no significant heterogeneity in effect (I^2^ 27.6, *P*Het = 0.25) when this cohort was excluded. In a random effects model the rs11121615 C-allele had an odds ratio for association with varicose veins of 1.22 (95%CI 1.09–1.36, *P* = 5.2 × 10^−4^) which increased to 1.28 (95%CI 1.22–1.35, *P* = 4.5 × 10^−22^) when the 23andMe cohort was excluded.

Because of the well clinically phenotyped nature of the NZ and NL cohorts a further analysis was conducted to determine if the rs111211615 C-allele showed any further stratified association with the clinical classification of CVD. In both cohorts the highest C-allele frequency was observed in those with CEAP C4-6. In the NZ cohort there was a significant difference between those with either self-declared varicose veins or varicose veins with or without edema (CEAP C3 or C2 respectively) and those with CEAP ≥ C4 (Fig. 1, C-allele frequency 0.35 versus 0.40 respectively, *P* = 0.02).

### Rs11121615 functional analysis

**Figure 4:**
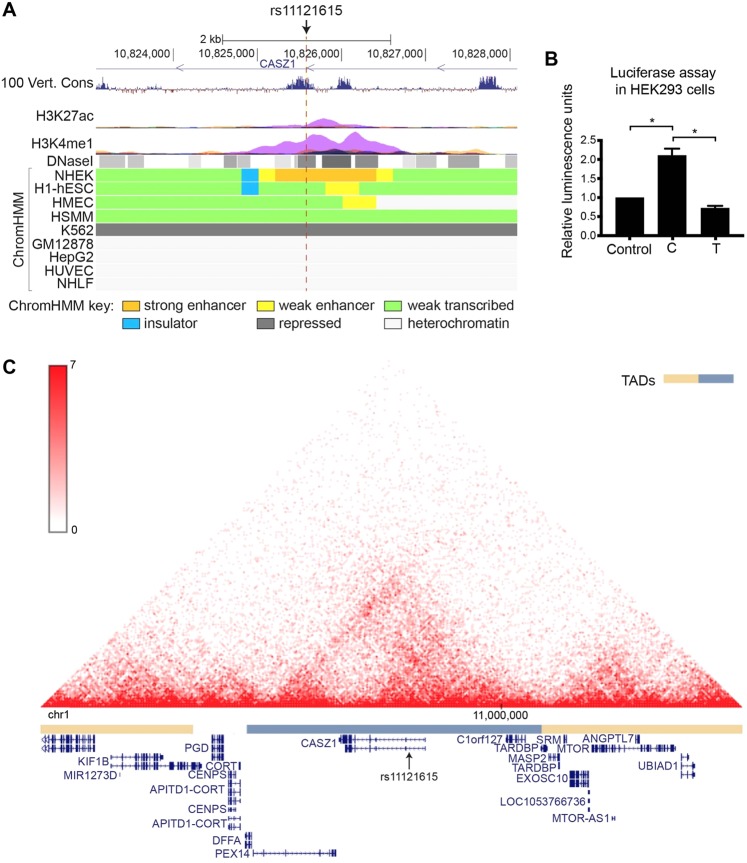
rs11121615 affects enhancer activity within a topologically associated domain (TAD) containing *CASZ1*. (**A**) UCSC snapshot showing the following tracks: UCSC genes, 100 vertebrates basewise conservation by PhyloP, ENCODE layered ChIP-Seq data for H3K27ac and H3K4me1 from seven cell lines (NHEK cells in purple), ENCODE DNase I hypersensitivity data, and 15-state chromatin state Segmentation by HMM (ChromHMM) for nine cell lines. hg19 chromosome 1 coordinates are shown. (**B**) Allele-specific enhancer activity for rs11121615 was assessed using a luciferase reporter assay in HEK293 cells. The rs11121615 T variant (major allele) did not harbour enhancer activity whereas the C variant (minor allele) did. The average of five biological replicates+/− the standard error of the mean is shown. Statistical significance was determined by a one-way ANOVA followed by a Tukey’s multiple comparisons test (*P < 0.0001). (**C**) Hi-C contact map in NHEK cells (normal human epidermal keratinocyte) at chr1:10140000-11450000 (hg19), obtained from the 3D genome browser (http://www.3dgenome.org). TADs, calculated using the directional bias method^13^, are indicated by alternating orange and blue bars. NCBI reference genes are annotated below.

The ability of rs11121615 to alter regulatory element activity was examined using publicly available ENCODE and Roadmap Epigenomics project data. In NHEK (normal human epidermal keratinocytes), the rs11121615 DNA region had a H3K27ac and H3K4me1 peak, DNase I hypersensitivity sites and was annotated as an enhancer by ChromHMM (Fig. 4A). It had documented enhancer functions in multiple other primary cell types (epithelial, brain, digestive, muscle, heart, lung, liver and pancreas) (Supplementary Table 2). Further, rs1121615 resides in a region that is conserved in vertebrates (Fig. 4A). Rs11121615 is predicted to alter the binding motif of the mouse zinc-finger transcription factors Zfp128, Zic1 and Zic2 (Supplementary Fig. 1), indicating it might affect gene regulation by altering the binding affinity of transcription factors.

To experimentally confirm whether the rs11121615 DNA region harbors enhancer activity, and to assess whether the SNP alters its activity, a luciferase assay was performed in HEK293 cells. The major (T) allele did not harbour enhancer activity, while the minor (C) allele showed significant activity (Fig. 4B). This data suggested that the risk allele of rs11121615 increases enhancer activity, and gives insight into the mechanism through which it could influence gene expression.

Whether rs11121615 regulates the expression of *CASZ1* and/or of genes further away was assessed using publicly available chromatin interaction (Hi-C) and expression quantitative trait loci (eQTL) data. Hi-C data in several cell lines indicates that rs11121615 is located within a topologically associated domain (TAD) spanning from *DFFA*/*PEX14* at the upstream boundary to *TARDBP* at the downstream boundary (Fig. 4C). The TAD boundaries were similar in all cell types for which Hi-C data is available (data not shown). Genes within the domain included *CASZ1* and *C1orf127* (an uncharacterized protein-coding gene) (Fig. 4C). Genes near the TAD boundaries, such as *DFFA* (DNA fragmentation factor subunit alpha), *PEX14* (a peroxisome membrane protein) and *TARDBP* (a DNA and RNA-binding protein that regulates transcription and splicing), are ‘housekeeping’ type of genes and have no known specific function in vascular-related cell types. Given that regulatory elements typically confine their activity within TADs, and TAD boundaries are enriched for housekeeping genes^12,13^, it is likely that the rs11121615-containing enhancer regulates the expression of *CASZ1* and/or *C1orf127*, and not of *DFFA/PEX14* or *TARDBP*.

eQTL data from GTEx (Genotype-Tissue Expression) and ASAP (Advanced Study of Aortic Pathology) were explored to identify eQTLs for rs11121615^14,15^. In GTEx, no significant eQTL for rs11121615 were present, while the ASAP study had two significant eQTLs for *CTNNBIP1* (*P* = 0.00082) and *TARDBP* (*P* = 0.013) in heart tissue. These genes are not located within the TAD however, and it is therefore unclear whether rs11121615 might regulate the expression of these genes.

## Discussion

This study validated by far the strongest previously reported genetic association with CVD^8,10^. Located within intron 1 of the *CASZ1* gene, the minor C-allele of rs11121615, had a significant positive association with CVD in all five of the independent populations examined to date. Of note, however, the fixed effect model suggested significant heterogeneity of effect, with the outlier population appearing to be the 23andMe cohort.

When considering results of case control genetic association studies, it is important to consider the quality of both case and control phenotyping. The 23andMe study, which was the first to report the *CASZ1* association, utilized self-declared case status, which does not inform the clinical severity of disease and is potentially prone to bias based on an individual’s perception of what constitutes a varicose vein. For example, in the 23andMe cohort the case prevalence in females was almost three times higher than males, an observation which does not match that reported in well-designed epidemiological studies^1^. Nor were cases and controls well matched for age in the study. Offsetting these issues was the very large sample size (over 20,000 cases) that was examined. In contrast, the UK Biobank dataset has over 10,000 varicose vein cases, with the advantage of improved phenotyping via electronic extraction of International Classification of Disease (ICD10) codes (I83; varicose veins of lower extremities), however, there was no clinical sub-classification of those with CVD. The Russian cohort reported by Shadrina and colleagues had cases confirmed by clinical examination and duplex ultrasound with all having visible varicose veins and reflux in either the great or small saphenous vein. The vast majority of this cohort (n = 590, 83.2%) had CEAP C2-3 classified disease.

In order to determine the possible impact of such self-declared case designation, we included a group of NZ participants who had self-reported as having varicose veins on a research recruitment health questionnaire (albeit with a degree of verification via visual observation of the patient’s lower limbs by a vascular sonographer). Encouragingly, these participants showed a similar varicose vein risk association for the rs11121615 C-allele to that of patients with CEAP C2-3 in both the NZ and Dutch studies, suggesting that, at least for this variant, the self-declared phenotype represents a valid association with varicose veins. Of note, however, those classified as having CEAP C1 venous disease did not show an association with the rs11121615 C-allele in either of the cohorts in this study, with allele frequencies being strikingly similar to venous disease-free controls. Given that CEAP C1 classified venous alterations are not considered to be varicose veins^11^, the rs11121615 association appears to represent a varicose vein specific effect. Moreover, this suggests that inclusion of CEAP C1 individuals in a case cohort, for example when case designation is collected via an unverified self-declared mechanism, would influence the resulting analysis towards the null hypothesis of no association.

We suggest that this is the possible explanation for the lower effect size observed in the 23andMe cohort. While the meta-analysis indicated a C-allele odds ratio of 1.2 when all cohorts were included, our data suggests that the association is more likely to be in the order of 1.3 for uncomplicated varicose veins (CEAP C2) and potentially as high as 1.5 for CVD with skin changes and venous ulcers (CEAP C4-6).

Rs11121615 is located in intron 1 of the *CASZ1* gene, and thus does not directly alter protein function. Our bioinformatic analysis of the region harboring rs11121615 suggested potential enhancer functions in multiple cell types. Importantly, we demonstrated that the varicose vein associated C-allele of this SNP confers a significant increase in enhancer activity. Chromatin interaction analysis suggested that *CASZ1* is a likely target of this enhancer regulation. *CASZ1* is expressed in human endothelial cells and drives the expression of Epidermal Growth Factor Like-Domain 7 *(EGFL7)*, which in turn drives the expression of the *RhoA* gene. RhoA mediates NF-κB dependent gene expression, including VEGFR2, and thereby stimulates angiogenesis and vascular assembly^16^. Taken together, our data supports the proposition that rs11121615 is a causal variant influencing risk of developing varicose veins via alterations in the transcription factor CASZ1, an upstream regulator of vascular structure.

The observation that the rs11121615 C-allele is more strongly associated with more severe forms of CVD, particularly venous ulceration, needs further investigation. If this proves to be the case, this variant may have utility identifying those individuals with varicose veins who are at greater risk of progression to more severe, higher classes of CVD.

## Methods

### Participants

All New Zealand participants were recruited from the Otago-Southland region of New Zealand’s South Island. Participants were either part of the Otago Vascular Genetics Research (OVGR) cohort^17^ or were patients referred for superficial venous disease assessment. OVGR participants completed a self-administered health questionnaire which included the question “Have you ever had, or been treated, for varicose veins”. All participants attended the recruitment facility in the local hospital, at which time the questionnaire was reviewed by experienced recruitment staff. Visual inspection of the limbs of those indicating that they had varicose veins was performed by an experienced vascular sonographer. Those with only telangiectasia or reticular veins were recoded as not having significant varicose veins. Patients referred for superficial venous disease assessment to the Otago Vascular Diagnostics Laboratory were also recruited. These patients underwent a formal assessment for lower limb superficial chronic venous disease, including full ultrasound examination, and were classified using the clinical classification matrix of the clinical, etiological, anatomical and pathological (CEAP) chronic venous disorders criteria^11^. For bilateral disease the class of the most severely affected limb was recorded. In brief, CEAP C0, no visible or palpable signs of venous disease; C1, telangiectasia or reticular veins; C2, varicose veins (VV), distinguished from reticular veins by a diameter of greater than or equal to 3 mm; C3, edema of venous origin; C4, skin hyperpigmentation or lipodermatosclerosis; C5, healed venous ulcer; C6, active venous ulceration.

All participants from the Netherlands (NL) were part of the Rotterdam Study^18^, and were clinically assessed using the same CEAP classification as the NZ venous disease cohort.

All participants gave written informed consent and all research was performed in accordance with relevant guidelines/ regulations. In New Zealand the study was approved by the Health and Disability Ethics Committee (New Zealand Ministry of Health), while in the Netherlands the Rotterdam study was approved by Erasmus Medical Center’s Medical Ethics Committee and by the review board of The Netherlands Ministry of Health, Welfare and Sports.

All participants had DNA extracted from an EDTA blood sample for genotyping. New Zealand samples were assessed using a Taqman assay (C_3092341_20, Thermofisher Scientific, MA, USA).

In the Rotterdam Study, DNA from whole blood was extracted following standard protocols^19^. Genome-wide SNP array data is available for the Rotterdam Study with details described by Hofman and colleagues^19^. SNP coverage was further expanded by imputation using the MACH software (http://csg.sph.umich.edu/abecasis/mach/index.html), with default parameters, and the 1000Genomes (GIANT Phase I version 3) as the reference panel. In total 30,072,738 markers were genotyped and/or imputed. From this data we extracted best guess genotypes for the rs11121615 SNP using GCTA^20^ with parameter defaults. The imputation quality score for this SNP was 0.76.

### Genetic association statistical analysis

The SNP-disease associations in the NZ and NL cohorts were determined using PLINK (http://pngu.mgh.harvard.edu/purcell/plink/)^21^. The Fisher’s exact test was used to test allelic associations. Logistic regression was used to determine interactions with covariates (age and sex). Meta-analysis was performed using fixed and random-effects modelling using the Comprehensive Meta-Analysis software package, version 3 (www.meta-analysis.com/). Effects sizes were reported as odds ratios (OR) and 95% confidence intervals. Inter-study heterogeneity was measured using the I2 statistic.

### Functional studies

#### Luciferase assay

Enhancer activity of the region spanning rs11121615 was assessed using a luciferase reporter assay in the human embryonic kidney cell line HEK293. A 2 kb region spanning rs11121615 was amplified from the 1000 Genomes sample HG00112 (heterozygous for rs11121615)^22^, cloned into the pCR®8/GW/TOPO entry vector and recombined into pGL4.23-GW (Addgene, MA, USA) using the Gateway® system (LR Clonase® Enzyme mix, Invitrogen, Thermofisher Scientific, MA, USA). Primers used to amplify the cloned regions can be found in Supplementary Table 4. The construct obtained was Sanger sequenced and contained the minor allele of rs11121615 (T). Site-directed mutagenesis (SDM) was carried out obtain a construct containing the major allele of rs11121615 (C). The SDM protocol used was adapted from the QuikChange II site-directed mutagenesis protocol (Agilent Technologies, CA, USA). PCR was performed using *PfuUltra* High-Fidelity DNA Polymerase (Agilent Technologies) using the primers listed in Supplementary Table 4. Parental plasmid DNA was digested with *Dpn*I (Agilent Technologies) and mutagenized plasmids were then transformed into *E. coli* TOP10 chemically competent cells. SDM was confirmed using Sanger sequencing. All plasmids used for transfections were isolated using the NucleoBond Xtra Midi endotoxin-free kit (Macherey-Nagel GmbH, Germany).

One day prior to transfection, HEK293 cells were seeded at 8 × 10^3^ cells per well in a 96-well plate in 200 μL DMEM supplemented with 10% fetal bovine serum (Moregate, NZ). Transfections were carried out using Lipofectamine 3000 (Invitrogen) with 100 ng of each pGL4.23 construct and 4 or 20 ng of pRL-TK (*Renilla*; Promega, WI, USA) per well. Luminescence was measured 48 hours post-transfection using the Dual Glo Luciferase Assay System (Promega), on a Perkin Elmer Victor X4 plate reader. Firefly luciferase was normalised to that of *Renilla* and expressed relative to pGL4.23 lacking a gateway cassette.

#### ChIP-seq, DNase I, Hi-C, eQTL data, and computational prediction of transcription factor binding motif affinity

Histone modification ChIP-seq, DNase I hypersensitivity and 15-state ChromHMM data was obtained from the ENCODE^23^ and Roadmap Epigenomics^24^ projects through the UCSC genome browser (https://genome.ucsc.edu/) and the 3DSNP database (http://cbportal.org/3dsnp/). Previously described Hi-C data in NHEK cells was obtained using the 3D genome browser (http://www.3dgenome.org). eQTL data were obtained from GTEx and ASAP. RegulomeDB (http://www.regulomedb.org) was used to predict alterations in transcription factor binding motifs.

## Supplementary information

Supplementary data

## Data Availability

The datasets generated during the current study are available from the corresponding author on reasonable request.
