# Supplementary material for: A variant of the castor zinc finger 1 (CASZ1) gene is differentially associated with the clinical classification of chronic venous disease
Source: Sci Rep. 2019 Sep 30;9:14011. doi: 10.1038/s41598-019-50586-2 (PMC6769056; doi:10.1038/s41598-019-50586-2)

**Supplementary data for:**

**A variant of the castor zinc finger 1 (*CASZ1*) gene is differentially associated with the clinical classification of chronic venous disease**

Gregory T Jones, Judith Marsman, Luba M Pardo, Tamar Nijsten, Marianne De Maeseneer, Vicky Phillips, Chi Lynch-Sutherland, Julia Horsfield, Jolanda Krysa & Andre M van Rij

**Supplementary Table 1.** Rs11121615 genotypes.

| **New Zealand Cohort** | **CC** | **CT** | **TT** |
| --- | --- | --- | --- |
| Controls | 193(0.103) | 752(0.401) | 931(0.496) |
| CEAP C1 | 7(0.069) | 47(0.483) | 43(0.448) |
| VV self-declared | 86(0.152) | 219(0.389) | 259(0.459) |
| CEAP C2-3 | 54(0.090) | 307(0.516) | 234(0.393) |
| CEAP C4 | 26(0.129) | 108(0.535) | 68(0.335) |
| CEAP C5-6 | 19(0.131) | 81(0.546) | 48(0.323) |
| VV (excluding CEAP C1) | 192(0.127) | 706(0.468) | 611(0.405) |
|  |  |  |  |
| **Dutch Cohort** | **CC** | **CT** | **TT** |
| Controls | 146(0.090) | 747(0.459) | 733(0.451) |
| CEAP C1 | 159(0.093) | 757(0.444) | 788(0.462) |
| CEAP C2-3 | 140(0.117) | 569(0.477) | 484(0.406) |
| CEAP C4-6 | 7(0.101) | 39(0.565) | 23(0.333) |
| CEAP C2-6 | 147(0.116) | 608(0.482) | 507(0.402) |

**Supplementary Table 2.** Rs11121615 g C-allele (unadjusted) odds ratios compared with controls.

| **New Zealand Cohort** | **OR** | **L95** | **U95** | **P-value** |
| --- | --- | --- | --- | --- |
| CEAP C1 | 1.03 | 0.74 | 1.44 | 0.84 |
| VV self-declared | 1.22 | 1.06 | 1.41 | 6.7E-3 |
| CEAP C2-3 | 1.23 | 1.06 | 1.43 | 5.8E-3 |
| CEAP C4 | 1.51 | 1.20 | 1.90 | 3.6E-4 |
| CEAP C5-6 | 1.56 | 1.20 | 2.01 | 7.1E-4 |
| VV (excluding CEAP C1) | 1.30 | 1.17 | 1.44 | 5.5E-7 |
|  |  |  |  |  |
| **Dutch Cohort** | **OR** | **L95** | **U95** | **P-value** |
| CEAP C1 | 0.98 | 0.89 | 1.09 | 0.721 |
| CEAP C2-3 | 1.18 | 1.05 | 1.32 | 0.004 |
| CEAP C4-6 | 1.33 | 0.94 | 1.89 | 0.112 |
| CEAP C2-6 | 1.18 | 1.06 | 1.32 | 0.003 |

**Supplementary Table 3. 3DSNP output of chromHMM states from ENCODE and Roadmap Epigenomics data.**

| Chromatin state | Cell type | Tissue | Description |
| --- | --- | --- | --- |
| Enhancers | BRN.GRM.MTRX | Brain | Brain Germinal Matrix |
| Enhancers | GI.CLN.MUC | Digestive | Colonic Mucosa |
| Enhancers | GI.DUO.MUC | Digestive | Duodenum Mucosa |
| Enhancers | GI.ESO | Digestive | Esophagus |
| Enhancers | GI.STMC.GAST | Digestive | Gastric |
| Enhancers | GI.RECT.MUC.29 | Digestive | Rectal Mucosa Donor 29 |
| Enhancers | GI.RECT.MUC.31 | Digestive | Rectal Mucosa Donor 31 |
| Enhancers | GI.STMC.MUC | Digestive | Stomach Mucosa |
| Enhancers | BRST.MYO | Epithelial | Breast Myoepithelial Primary Cells |
| Enhancers | SKIN.PEN.FRSK.KER.02 | Epithelial | Foreskin Keratinocyte Primary Cells skin02 |
| Enhancers | SKIN.PEN.FRSK.KER.03 | Epithelial | Foreskin Keratinocyte Primary Cells skin03 |
| Enhancers | HRT.FET | Heart | Fetal Heart |
| Enhancers | HRT.VENT.L | Heart | Left Ventricle |
| Enhancers | HRT.ATR.R | Heart | Right Atrium |
| Enhancers | HRT.VNT.R | Heart | Right Ventricle |
| Enhancers | LNG | Lung | Lung |
| Enhancers | GI.CLN.SM.MUS | Muscle | Colon Smooth Muscle |
| Enhancers | MUS.LEG.FET | Muscle | Fetal Muscle Leg |
| Enhancers | MUS.PSOAS | Muscle | Psoas Muscle |
| Enhancers | MUS.SKLT.M | Muscle | Skeletal Muscle Male |
| Enhancers | MUS.SKLT.F | Muscle | Skeletal Muscle Female |
| Enhancers | GI.STMC.MUS | Muscle | Stomach Smooth Muscle |
| Enhancers | LIV.ADLT | Other | Liver |
| Enhancers | LNG.FET | Other | Fetal Lung |
| Enhancers | PANC | Other | Pancreas |
| Flanking Active TSS | SKIN.NHEK | Skin | NHEK-Epidermal Keratinocyte Primary Cells |

**Supplementary Table 4. Primer sequences.**

| **Primer name** | **Sequence 5’ to 3’** |
| --- | --- |
| rs11121615 forward | GATAGATTAGGGAGGGCGGG |
| rs11121615 reverse | TTGTGACATCTGAGCCCTGT |
| rs11121615 SDM F | GAATACCTGCTGCGTTGGGCGTACCCGTC |
| rs11121615 SDM R | GACGGGTACGCCCAACGCAGCAGGTATTC |


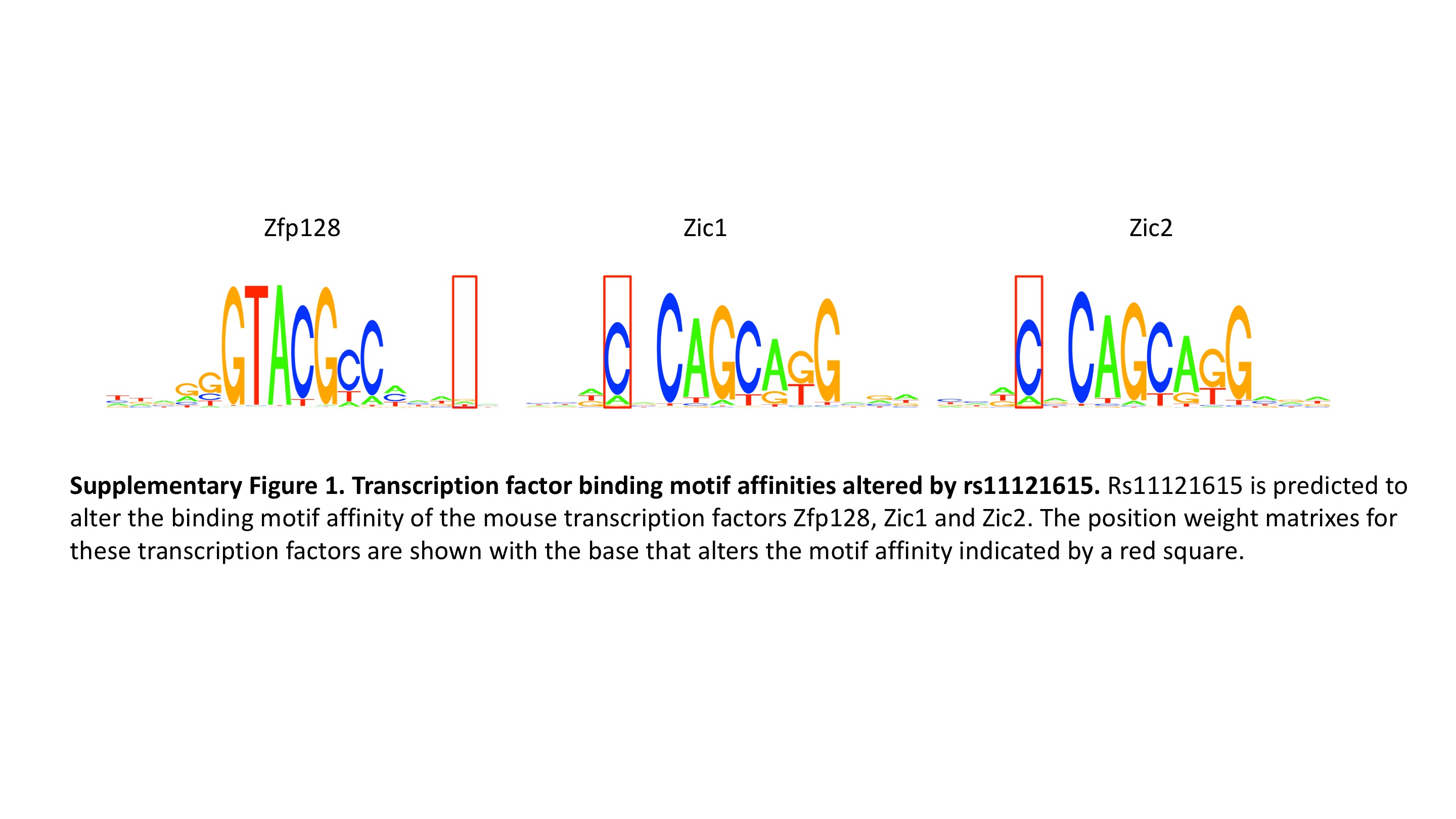

Supplement: Supplementary file 1 — Supplementary data [file 41598_2019_50586_MOESM1_ESM.docx]
